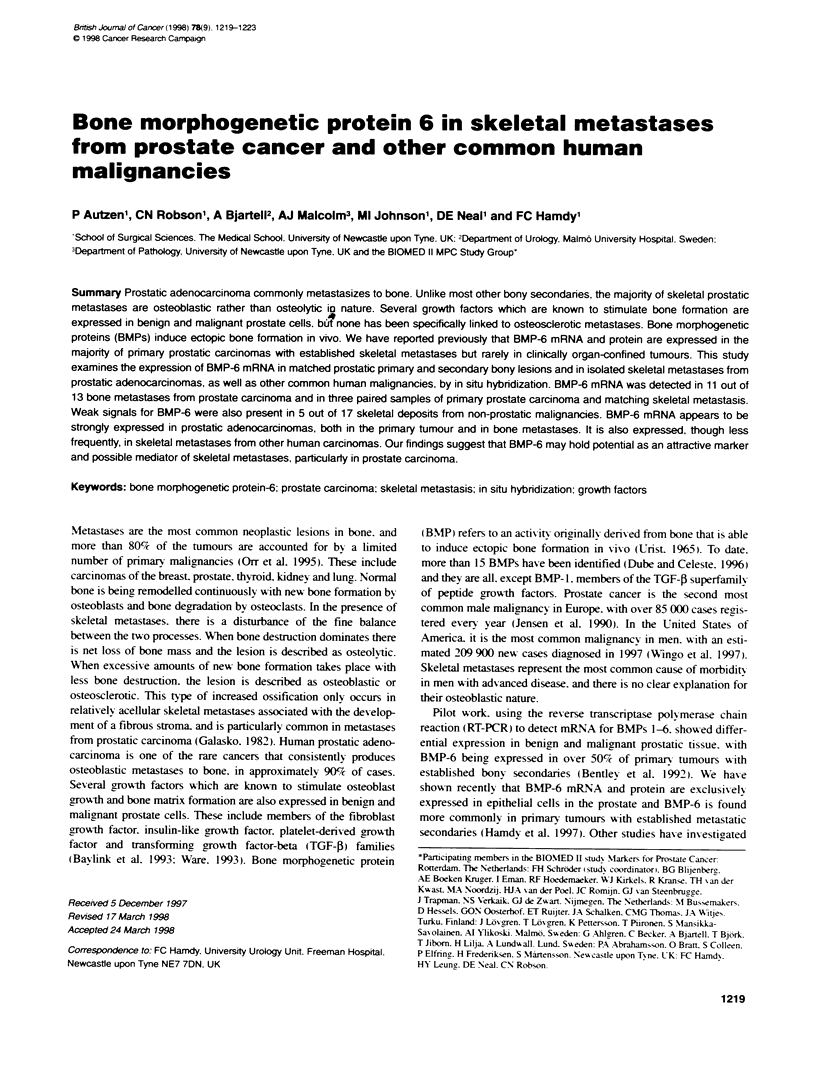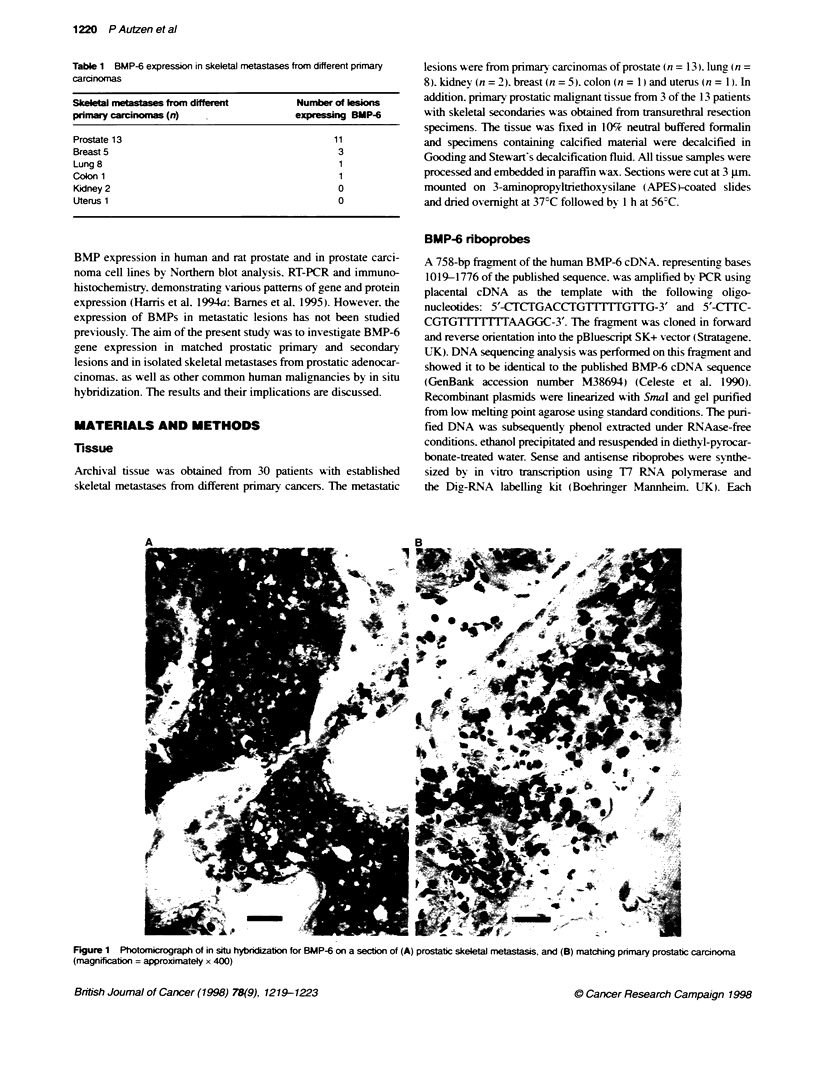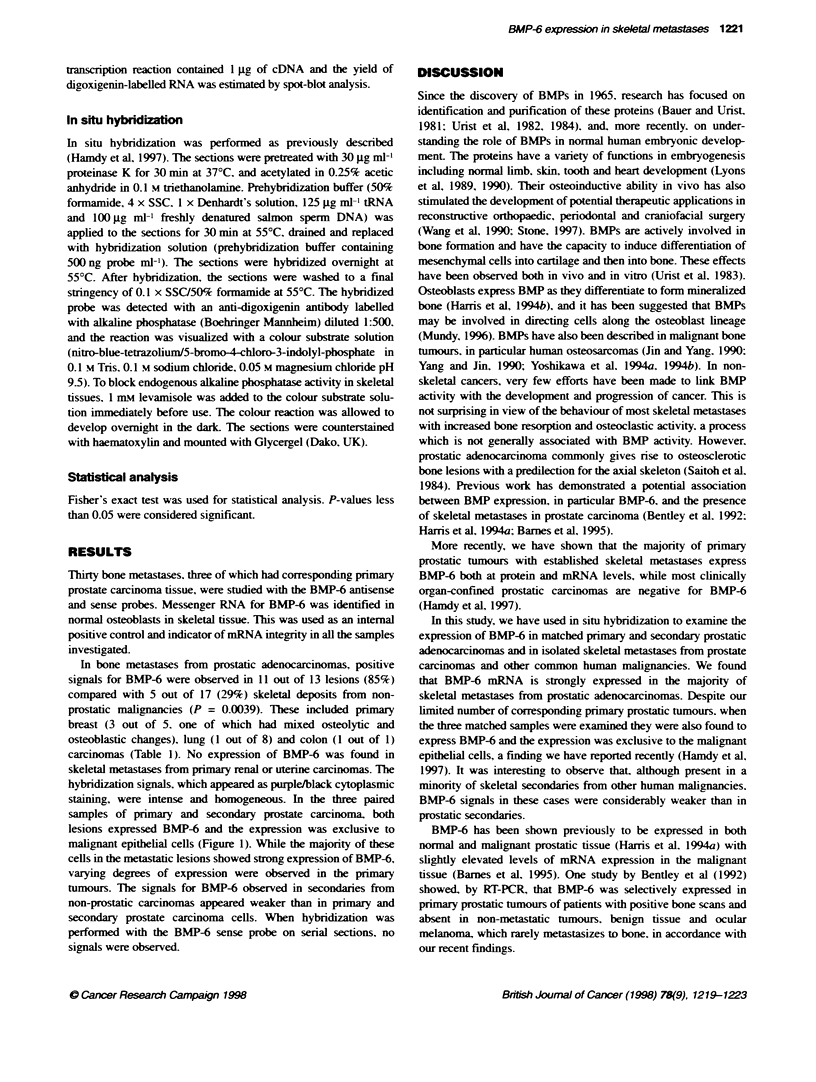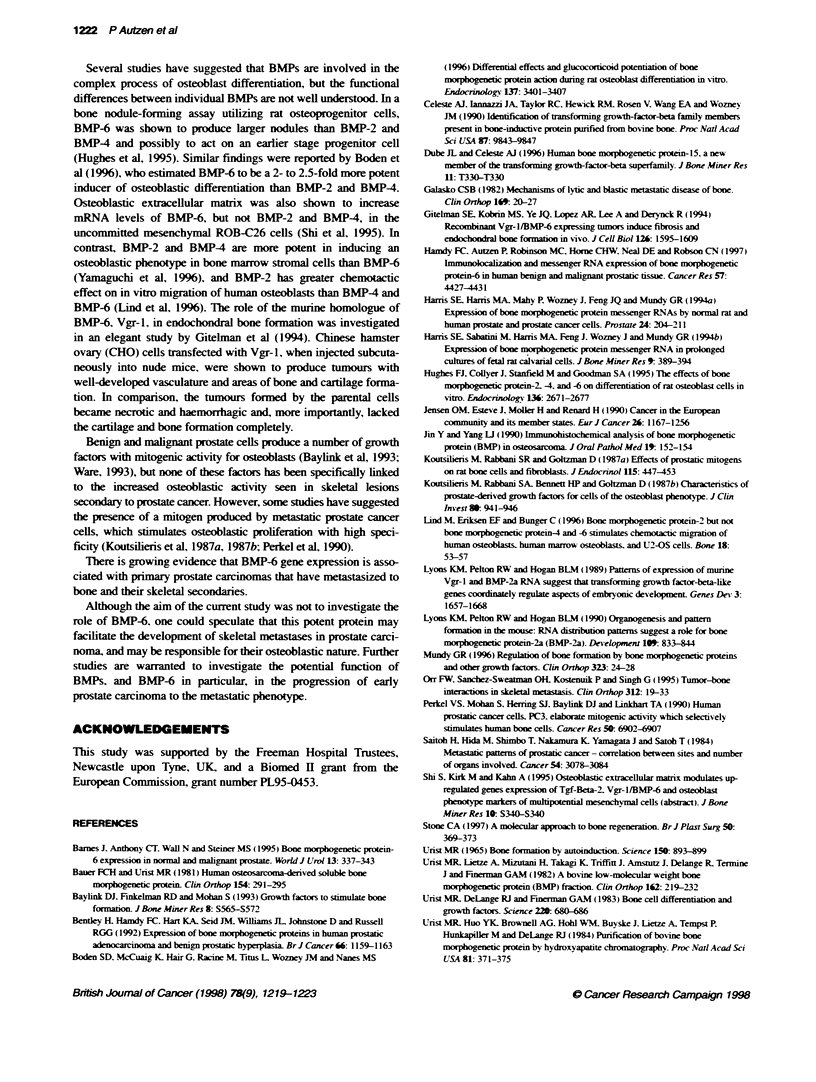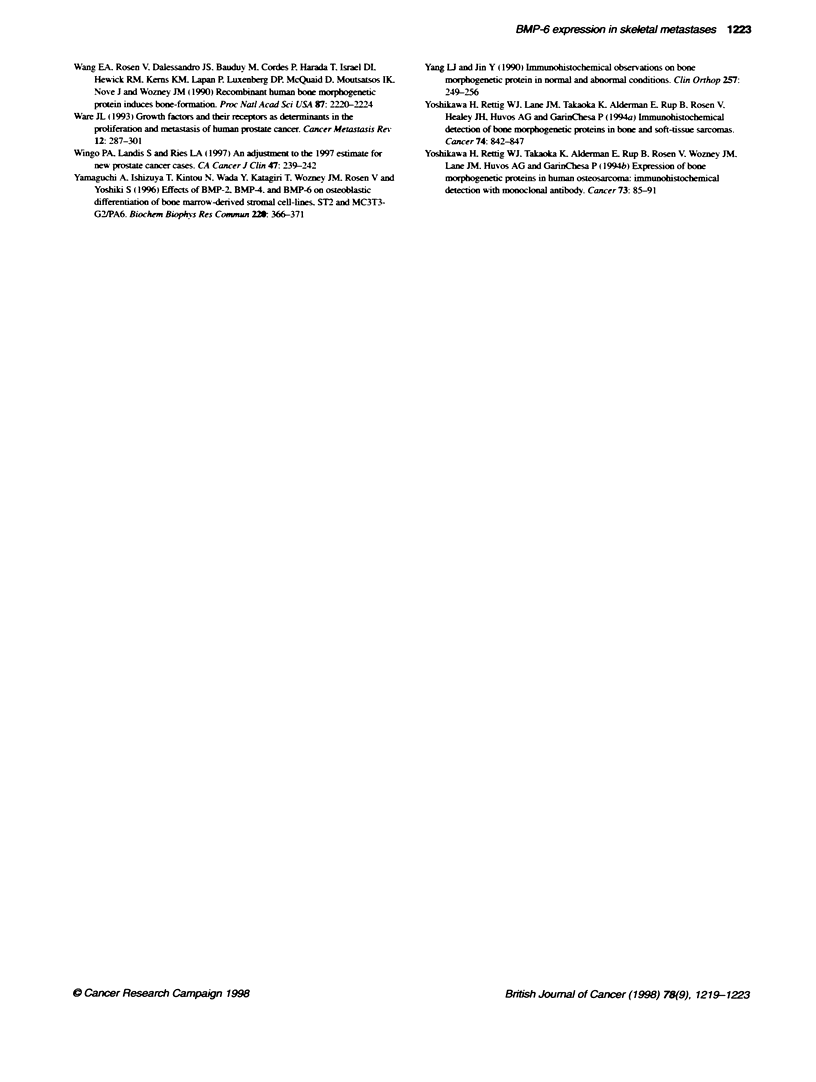# Bone morphogenetic protein 6 in skeletal metastases from prostate cancer and other common human malignancies.

**DOI:** 10.1038/bjc.1998.658

**Published:** 1998-11

**Authors:** P. Autzen, C. N. Robson, A. Bjartell, A. J. Malcolm, M. I. Johnson, D. E. Neal, F. C. Hamdy

**Affiliations:** School of Surgical Sciences, The Medical School, University of Newcastle upon Tyne, UK.

## Abstract

**Images:**


British Journal of Cancer (1998) 78(9). 1219–1223
© 1998 Cancer Research Campaign

### Bone morphogenetic protein 6 in skeletal metastases from prostate cancer and other common human malignancies

**P Autzen[1], CN Robson[1], A Bjartell[2], AJ Malcolm[3], MI Johnson[1], DE Neal[1] and FC Hamdy[1]**

[1]School of Surgical Sciences. The Medical School. University of Newcastle upon Tyne. UK: [2]Department of Urology. Malmö University Hospital. Sweden: [3]Department of Pathology, University of Newcastle upon Tyne. UK and the BIOMED II MPC Study Group*

**Summary** Prostatic adenocarcinoma commonly metastasizes to bone. Unlike most other bony secondaries, the majority of skeletal prostatic metastases are osteoblastic rather than osteolytic in nature. Several growth factors which are known to stimulate bone formation are expressed in benign and malignant prostate cells, but none has been specifically linked to osteosclerotic metastases. Bone morphogenetic proteins (BMPs) induce ectopic bone formation in vivo. We have reported previously that BMP-6 mRNA and protein are expressed in the majority of primary prostatic carcinomas with established skeletal metastases but rarely in clinically organ-confined tumours. This study examines the expression of BMP-6 mRNA in matched prostatic primary and secondary bony lesions and in isolated skeletal metastases from prostatic adenocarcinomas, as well as other common human malignancies, by in situ hybridization. BMP-6 mRNA was detected in 11 out of 13 bone metastases from prostate carcinoma and in three paired samples of primary prostate carcinoma and matching skeletal metastasis. Weak signals for BMP-6 were also present in 5 out of 17 skeletal deposits from non-prostatic malignancies. BMP-6 mRNA appears to be strongly expressed in prostatic adenocarcinomas, both in the primary tumour and in bone metastases. It is also expressed, though less frequently, in skeletal metastases from other human carcinomas. Our findings suggest that BMP-6 may hold potential as an attractive marker and possible mediator of skeletal metastases, particularly in prostate carcinoma.

**Keywords:** bone morphogenetic protein-6; prostate carcinoma; skeletal metastasis; in situ hybridization; growth factors

Metastases are the most common neoplastic lesions in bone, and more than 80% of the tumours are accounted for by a limited number of primary malignancies (Orr et al. 1995). These include carcinomas of the breast, prostate, thyroid, kidney and lung. Normal bone is being remodelled continuously with new bone formation by osteoblasts and bone degradation by osteoclasts. In the presence of skeletal metastases, there is a disturbance of the fine balance between the two processes. When bone destruction dominates there is net loss of bone mass and the lesion is described as osteolytic. When excessive amounts of new bone formation takes place with less bone destruction, the lesion is described as osteoblastic or osteosclerotic. This type of increased ossification only occurs in relatively acellular skeletal metastases associated with the development of a fibrous stroma, and is particularly common in metastases from prostatic carcinoma (Galasko, 1982). Human prostatic adenocarcinoma is one of the rare cancers that consistently produces osteoblastic metastases to bone, in approximately 90% of cases. Several growth factors which are known to stimulate osteoblast growth and bone matrix formation are also expressed in benign and malignant prostate cells. These include members of the fibroblast growth factor, insulin-like growth factor, platelet-derived growth factor and transforming growth factor-beta (TGF-β) families (Baylink et al. 1993; Ware, 1993). Bone morphogenetic protein (BMP) refers to an activity originally derived from bone that is able to induce ectopic bone formation in vivo (Urist, 1965). To date, more than 15 BMPs have been identified (Dube and Celeste, 1996) and they are all, except BMP-1, members of the TGF-β superfamily of peptide growth factors. Prostate cancer is the second most common male malignancy in Europe, with over 85 000 cases registered every year (Jensen et al. 1990). In the United States of America, it is the most common malignancy in men, with an estimated 209 900 new cases diagnosed in 1997 (Wingo et al. 1997). Skeletal metastases represent the most common cause of morbidity in men with advanced disease, and there is no clear explanation for their osteoblastic nature.

Pilot work, using the reverse transcriptase polymerase chain reaction (RT-PCR) to detect mRNA for BMPs 1–6, showed differential expression in benign and malignant prostatic tissue, with BMP-6 being expressed in over 50% of primary tumours with established bony secondaries (Bentley et al. 1992). We have shown recently that BMP-6 mRNA and protein are exclusively expressed in epithelial cells in the prostate and BMP-6 is found more commonly in primary tumours with established metastatic secondaries (Hamdy et al. 1997). Other studies have investigated

*Received 5 December 1997*
*Revised 17 March 1998*
*Accepted 24 March 1998*

*Correspondence to:* FC Hamdy, University Urology Unit, Freeman Hospital, Newcastle upon Tyne NE7 7DN, UK

*Participating members in the BIOMED II study Markers for Prostate Cancer: Rotterdam, The Netherlands: FH Schröder (study coordinator), BG Blijenberg, AE Boeken Kruger, I Eman, RF Hoedemaeker, WJ Kirkels, R Kranse, TH van der Kwast, MA Noordzij, HJA van der Poel, JC Romijn, GJ van Steenbrugge, J Trapman, NS Verkaik, GJ de Zwart. Nijmegen, The Netherlands: M Bussemakers, D Hessels, GON Oosterhof, ET Ruijter, JA Schalken, CMG Thomas, JA Witjes. Turku, Finland: J Lövgren, T Lövgren, K Pettersson, T Piironen, S Mansikka-Savolainen, AI Ylikoski. Malmö, Sweden: G Ahlgren, C Becker, A Bjartell, T Björk, T Jiborn, H Lilja, A Lundwall. Lund, Sweden: PA Abrahamsson, O Bratt, S Colleen, P Elfring, H Frederiksen, S Mårtensson. Newcastle upon Tyne, UK: FC Hamdy, HY Leung, DE Neal, CN Robson.

**Table 1** BMP-6 expression in skeletal metastases from different primary carcinomas

| Skeletal metastases from different primary carcinomas (n) | Number of lesions expressing BMP-6 |
|---|---|
| Prostate 13 | 11 |
| Breast 5 | 3 |
| Lung 8 | 1 |
| Colon 1 | 1 |
| Kidney 2 | 0 |
| Uterus 1 | 0 |

BMP expression in human and rat prostate and in prostate carcinoma cell lines by Northern blot analysis, RT-PCR and immunohistochemistry, demonstrating various patterns of gene and protein expression (Harris et al. 1994*a*; Barnes et al. 1995). However, the expression of BMPs in metastatic lesions has not been studied previously. The aim of the present study was to investigate BMP-6 gene expression in matched prostatic primary and secondary lesions and in isolated skeletal metastases from prostatic adenocarcinomas, as well as other common human malignancies by in situ hybridization. The results and their implications are discussed.

### MATERIALS AND METHODS

### Tissue

Archival tissue was obtained from 30 patients with established skeletal metastases from different primary cancers. The metastatic lesions were from primary carcinomas of prostate ($n$ = 13), lung ($n$ = 8), kidney ($n$ = 2), breast ($n$ = 5), colon ($n$ = 1) and uterus ($n$ = 1). In addition, primary prostatic malignant tissue from 3 of the 13 patients with skeletal secondaries was obtained from transurethral resection specimens. The tissue was fixed in 10% neutral buffered formalin and specimens containing calcified material were decalcified in Gooding and Stewart's decalcification fluid. All tissue samples were processed and embedded in paraffin wax. Sections were cut at 3 μm, mounted on 3-aminopropyltriethoxysilane (APES)-coated slides and dried overnight at 37°C followed by 1 h at 56°C.

### BMP-6 riboprobes

A 758-bp fragment of the human BMP-6 cDNA, representing bases 1019–1776 of the published sequence, was amplified by PCR using placental cDNA as the template with the following oligonucleotides: 5′-CTCTGACCTGTTTTTGTTG-3′ and 5′-CTTCCGTGTTTTTTTTAAGGC-3′. The fragment was cloned in forward and reverse orientation into the pBluescript SK+ vector (Stratagene, UK). DNA sequencing analysis was performed on this fragment and showed it to be identical to the published BMP-6 cDNA sequence (GenBank accession number M38694) (Celeste et al. 1990). Recombinant plasmids were linearized with *Sma*I and gel purified from low melting point agarose using standard conditions. The purified DNA was subsequently phenol extracted under RNAase-free conditions, ethanol precipitated and resuspended in diethyl-pyrocarbonate-treated water. Sense and antisense riboprobes were synthesized by in vitro transcription using T7 RNA polymerase and the Dig-RNA labelling kit (Boehringer Mannheim, UK). Each

**Figure 1** Photomicrograph of in situ hybridization for BMP-6 on a section of (**A**) prostatic skeletal metastasis, and (**B**) matching primary prostatic carcinoma (magnification = approximately × 400)

© Cancer Research Campaign 1998

transcription reaction contained 1 μg of cDNA and the yield of digoxigenin-labelled RNA was estimated by spot-blot analysis.

### In situ hybridization

In situ hybridization was performed as previously described (Hamdy et al, 1997). The sections were pretreated with 30 μg $ml^{-1}$ proteinase K for 30 min at 37°C, and acetylated in 0.25% acetic anhydride in 0.1 M triethanolamine. Prehybridization buffer (50% formamide, 4 × SSC, 1 × Denhardt's solution, 125 μg $ml^{-1}$ tRNA and 100 μg $ml^{-1}$ freshly denatured salmon sperm DNA) was applied to the sections for 30 min at 55°C, drained and replaced with hybridization solution (prehybridization buffer containing 500 ng probe $ml^{-1}$). The sections were hybridized overnight at 55°C. After hybridization, the sections were washed to a final stringency of 0.1 × SSC/50% formamide at 55°C. The hybridized probe was detected with an anti-digoxigenin antibody labelled with alkaline phosphatase (Boehringer Mannheim) diluted 1:500, and the reaction was visualized with a colour substrate solution (nitro-blue-tetrazolium/5-bromo-4-chloro-3-indolyl-phosphate in 0.1 M Tris, 0.1 M sodium chloride, 0.05 M magnesium chloride pH 9.5). To block endogenous alkaline phosphatase activity in skeletal tissues, 1 mM levamisole was added to the colour substrate solution immediately before use. The colour reaction was allowed to develop overnight in the dark. The sections were counterstained with haematoxylin and mounted with Glycergel (Dako, UK).

### Statistical analysis

Fisher's exact test was used for statistical analysis. *P*-values less than 0.05 were considered significant.

### RESULTS

Thirty bone metastases, three of which had corresponding primary prostate carcinoma tissue, were studied with the BMP-6 antisense and sense probes. Messenger RNA for BMP-6 was identified in normal osteoblasts in skeletal tissue. This was used as an internal positive control and indicator of mRNA integrity in all the samples investigated.

In bone metastases from prostatic adenocarcinomas, positive signals for BMP-6 were observed in 11 out of 13 lesions (85%) compared with 5 out of 17 (29%) skeletal deposits from non-prostatic malignancies ($P = 0.0039$). These included primary breast (3 out of 5, one of which had mixed osteolytic and osteoblastic changes), lung (1 out of 8) and colon (1 out of 1) carcinomas (Table 1). No expression of BMP-6 was found in skeletal metastases from primary renal or uterine carcinomas. The hybridization signals, which appeared as purple/black cytoplasmic staining, were intense and homogeneous. In the three paired samples of primary and secondary prostate carcinoma, both lesions expressed BMP-6 and the expression was exclusive to malignant epithelial cells (Figure 1). While the majority of these cells in the metastatic lesions showed strong expression of BMP-6, varying degrees of expression were observed in the primary tumours. The signals for BMP-6 observed in secondaries from non-prostatic carcinomas appeared weaker than in primary and secondary prostate carcinoma cells. When hybridization was performed with the BMP-6 sense probe on serial sections, no signals were observed.

### DISCUSSION

Since the discovery of BMPs in 1965, research has focused on identification and purification of these proteins (Bauer and Urist, 1981; Urist et al, 1982, 1984), and, more recently, on understanding the role of BMPs in normal human embryonic development. The proteins have a variety of functions in embryogenesis including normal limb, skin, tooth and heart development (Lyons et al, 1989, 1990). Their osteoinductive ability in vivo has also stimulated the development of potential therapeutic applications in reconstructive orthopaedic, periodontal and craniofacial surgery (Wang et al, 1990; Stone, 1997). BMPs are actively involved in bone formation and have the capacity to induce differentiation of mesenchymal cells into cartilage and then into bone. These effects have been observed both in vivo and in vitro (Urist et al, 1983). Osteoblasts express BMP as they differentiate to form mineralized bone (Harris et al, 1994*b*), and it has been suggested that BMPs may be involved in directing cells along the osteoblast lineage (Mundy, 1996). BMPs have also been described in malignant bone tumours, in particular human osteosarcomas (Jin and Yang, 1990; Yang and Jin, 1990; Yoshikawa et al, 1994*a*, 1994*b*). In non-skeletal cancers, very few efforts have been made to link BMP activity with the development and progression of cancer. This is not surprising in view of the behaviour of most skeletal metastases with increased bone resorption and osteoclastic activity, a process which is not generally associated with BMP activity. However, prostatic adenocarcinoma commonly gives rise to osteosclerotic bone lesions with a predilection for the axial skeleton (Saitoh et al, 1984). Previous work has demonstrated a potential association between BMP expression, in particular BMP-6, and the presence of skeletal metastases in prostate carcinoma (Bentley et al, 1992; Harris et al, 1994*a*; Barnes et al, 1995).

More recently, we have shown that the majority of primary prostatic tumours with established skeletal metastases express BMP-6 both at protein and mRNA levels, while most clinically organ-confined prostatic carcinomas are negative for BMP-6 (Hamdy et al, 1997).

In this study, we have used in situ hybridization to examine the expression of BMP-6 in matched primary and secondary prostatic adenocarcinomas and in isolated skeletal metastases from prostate carcinomas and other common human malignancies. We found that BMP-6 mRNA is strongly expressed in the majority of skeletal metastases from prostatic adenocarcinomas. Despite our limited number of corresponding primary prostatic tumours, when the three matched samples were examined they were also found to express BMP-6 and the expression was exclusive to the malignant epithelial cells, a finding we have reported recently (Hamdy et al, 1997). It was interesting to observe that, although present in a minority of skeletal secondaries from other human malignancies, BMP-6 signals in these cases were considerably weaker than in prostatic secondaries.

BMP-6 has been shown previously to be expressed in both normal and malignant prostatic tissue (Harris et al, 1994*a*) with slightly elevated levels of mRNA expression in the malignant tissue (Barnes et al, 1995). One study by Bentley et al (1992) showed, by RT-PCR, that BMP-6 was selectively expressed in primary prostatic tumours of patients with positive bone scans and absent in non-metastatic tumours, benign tissue and ocular melanoma, which rarely metastasizes to bone, in accordance with our recent findings.

© Cancer Research Campaign 1998

Several studies have suggested that BMPs are involved in the complex process of osteoblast differentiation, but the functional differences between individual BMPs are not well understood. In a bone nodule-forming assay utilizing rat osteoprogenitor cells, BMP-6 was shown to produce larger nodules than BMP-2 and BMP-4 and possibly to act on an earlier stage progenitor cell (Hughes et al, 1995). Similar findings were reported by Boden et al (1996), who estimated BMP-6 to be a 2- to 2.5-fold more potent inducer of osteoblastic differentiation than BMP-2 and BMP-4. Osteoblastic extracellular matrix was also shown to increase mRNA levels of BMP-6, but not BMP-2 and BMP-4, in the uncommitted mesenchymal ROB-C26 cells (Shi et al, 1995). In contrast, BMP-2 and BMP-4 are more potent in inducing an osteoblastic phenotype in bone marrow stromal cells than BMP-6 (Yamaguchi et al, 1996), and BMP-2 has greater chemotactic effect on in vitro migration of human osteoblasts than BMP-4 and BMP-6 (Lind et al, 1996). The role of the murine homologue of BMP-6, Vgr-1, in endochondral bone formation was investigated in an elegant study by Gitelman et al (1994). Chinese hamster ovary (CHO) cells transfected with Vgr-1, when injected subcutaneously into nude mice, were shown to produce tumours with well-developed vasculature and areas of bone and cartilage formation. In comparison, the tumours formed by the parental cells became necrotic and haemorrhagic and, more importantly, lacked the cartilage and bone formation completely.

Benign and malignant prostate cells produce a number of growth factors with mitogenic activity for osteoblasts (Baylink et al, 1993; Ware, 1993), but none of these factors has been specifically linked to the increased osteoblastic activity seen in skeletal lesions secondary to prostate cancer. However, some studies have suggested the presence of a mitogen produced by metastatic prostate cancer cells, which stimulates osteoblastic proliferation with high specificity (Koutsilieris et al, 1987*a*, 1987*b*; Perkel et al, 1990).

There is growing evidence that BMP-6 gene expression is associated with primary prostate carcinomas that have metastasized to bone and their skeletal secondaries.

Although the aim of the current study was not to investigate the role of BMP-6, one could speculate that this potent protein may facilitate the development of skeletal metastases in prostate carcinoma, and may be responsible for their osteoblastic nature. Further studies are warranted to investigate the potential function of BMPs, and BMP-6 in particular, in the progression of early prostate carcinoma to the metastatic phenotype.

### ACKNOWLEDGEMENTS

This study was supported by the Freeman Hospital Trustees, Newcastle upon Tyne, UK, and a Biomed II grant from the European Commission, grant number PL95-0453.

### REFERENCES

Barnes J, Anthony CT, Wall N and Steiner MS (1995) Bone morphogenetic protein-6 expression in normal and malignant prostate. *World J Urol* **13**: 337–343

Bauer FCH and Urist MR (1981) Human osteosarcoma-derived soluble bone morphogenetic protein. *Clin Orthop* **154**: 291–295

Baylink DJ, Finkelman RD and Mohan S (1993) Growth factors to stimulate bone formation. *J Bone Miner Res* **8**: S565–S572

Bentley H, Hamdy FC, Hart KA, Seid JM, Williams JL, Johnstone D and Russell RGG (1992) Expression of bone morphogenetic proteins in human prostatic adenocarcinoma and benign prostatic hyperplasia. *Br J Cancer* **66**: 1159–1163

Boden SD, McCuaig K, Hair G, Racine M, Titus L, Wozney JM and Nanes MS (1996) Differential effects and glucocorticoid potentiation of bone morphogenetic protein action during rat osteoblast differentiation in vitro. *Endocrinology* **137**: 3401–3407

Celeste AJ, Iannazzi JA, Taylor RC, Hewick RM, Rosen V, Wang EA and Wozney JM (1990) Identification of transforming growth-factor-beta family members present in bone-inductive protein purified from bovine bone. *Proc Natl Acad Sci USA* **87**: 9843–9847

Dube JL and Celeste AJ (1996) Human bone morphogenetic protein-15, a new member of the transforming growth-factor-beta superfamily. *J Bone Miner Res* **11**: T330–T330

Galasko CSB (1982) Mechanisms of lytic and blastic metastatic disease of bone. *Clin Orthop* **169**: 20–27

Gitelman SE, Kobrin MS, Ye JQ, Lopez AR, Lee A and Derynck R (1994) Recombinant Vgr-1/BMP-6 expressing tumors induce fibrosis and endochondral bone formation in vivo. *J Cell Biol* **126**: 1595–1609

Hamdy FC, Autzen P, Robinson MC, Horne CHW, Neal DE and Robson CN (1997) Immunolocalization and messenger RNA expression of bone morphogenetic protein-6 in human benign and malignant prostatic tissue. *Cancer Res* **57**: 4427–4431

Harris SE, Harris MA, Mahy P, Wozney J, Feng JQ and Mundy GR (1994*a*) Expression of bone morphogenetic protein messenger RNAs by normal rat and human prostate and prostate cancer cells. *Prostate* **24**: 204–211

Harris SE, Sabatini M, Harris MA, Feng J, Wozney J and Mundy GR (1994*b*) Expression of bone morphogenetic protein messenger RNA in prolonged cultures of fetal rat calvarial cells. *J Bone Miner Res* **9**: 389–394

Hughes FJ, Collyer J, Stanfield M and Goodman SA (1995) The effects of bone morphogenetic protein-2, -4, and -6 on differentiation of rat osteoblast cells in vitro. *Endocrinology* **136**: 2671–2677

Jensen OM, Esteve J, Moller H and Renard H (1990) Cancer in the European community and its member states. *Eur J Cancer* **26**: 1167–1256

Jin Y and Yang LJ (1990) Immunohistochemical analysis of bone morphogenetic protein (BMP) in osteosarcoma. *J Oral Pathol Med* **19**: 152–154

Koutsilieris M, Rabbani SR and Goltzman D (1987*a*) Effects of prostatic mitogens on rat bone cells and fibroblasts. *J Endocrinol* **115**: 447–453

Koutsilieris M, Rabbani SA, Bennett HP and Goltzman D (1987*b*) Characteristics of prostate-derived growth factors for cells of the osteoblast phenotype. *J Clin Invest* **80**: 941–946

Lind M, Eriksen EF and Bunger C (1996) Bone morphogenetic protein-2 but not bone morphogenetic protein-4 and -6 stimulates chemotactic migration of human osteoblasts, human marrow osteoblasts, and U2-OS cells. *Bone* **18**: 53–57

Lyons KM, Pelton RW and Hogan BLM (1989) Patterns of expression of murine Vgr-1 and BMP-2a RNA suggest that transforming growth factor-beta-like genes coordinately regulate aspects of embryonic development. *Genes Dev* **3**: 1657–1668

Lyons KM, Pelton RW and Hogan BLM (1990) Organogenesis and pattern formation in the mouse: RNA distribution patterns suggest a role for bone morphogenetic protein-2a (BMP-2a). *Development* **109**: 833–844

Mundy GR (1996) Regulation of bone formation by bone morphogenetic proteins and other growth factors. *Clin Orthop* **323**: 24–28

Orr FW, Sanchez-Sweatman OH, Kostenuik P and Singh G (1995) Tumor–bone interactions in skeletal metastasis. *Clin Orthop* **312**: 19–33

Perkel VS, Mohan S, Herring SJ, Baylink DJ and Linkhart TA (1990) Human prostatic cancer cells, PC3, elaborate mitogenic activity which selectively stimulates human bone cells. *Cancer Res* **50**: 6902–6907

Saitoh H, Hida M, Shimbo T, Nakamura K, Yamagata J and Satoh T (1984) Metastatic patterns of prostatic cancer – correlation between sites and number of organs involved. *Cancer* **54**: 3078–3084

Shi S, Kirk M and Kahn A (1995) Osteoblastic extracellular matrix modulates up-regulated genes expression of Tgf-Beta-2, Vgr-1/BMP-6 and osteoblast phenotype markers of multipotential mesenchymal cells (abstract). *J Bone Miner Res* **10**: S340–S340

Stone CA (1997) A molecular approach to bone regeneration. *Br J Plast Surg* **50**: 369–373

Urist MR (1965) Bone formation by autoinduction. *Science* **150**: 893–899

Urist MR, Lietze A, Mizutani H, Takagi K, Triffitt J, Amstutz J, Delange R, Termine J and Finerman GAM (1982) A bovine low-molecular weight bone morphogenetic protein (BMP) fraction. *Clin Orthop* **162**: 219–232

Urist MR, DeLange RJ and Finerman GAM (1983) Bone cell differentiation and growth factors. *Science* **220**: 680–686

Urist MR, Huo YK, Brownell AG, Hohl WM, Buyske J, Lietze A, Tempst P, Hunkapiller M and DeLange RJ (1984) Purification of bovine bone morphogenetic protein by hydroxyapatite chromatography. *Proc Natl Acad Sci USA* **81**: 371–375

© Cancer Research Campaign 1998

Wang EA. Rosen V. Dalessandro JS. Bauduy M. Cordes P. Harada T. Israel DI. Hewick RM. Kerns KM. Lapan P. Luxenberg DP. McQuaid D. Moutsatsos IK. Nove J and Wozney JM (1990) Recombinant human bone morphogenetic protein induces bone-formation. *Proc Natl Acad Sci USA* **87**: 2220–2224

Ware JL (1993) Growth factors and their receptors as determinants in the proliferation and metastasis of human prostate cancer. *Cancer Metastasis Rev* **12**: 287–301

Wingo PA. Landis S and Ries LA (1997) An adjustment to the 1997 estimate for new prostate cancer cases. *CA Cancer J Clin* **47**: 239–242

Yamaguchi A. Ishizuya T. Kintou N. Wada Y. Katagiri T. Wozney JM. Rosen V and Yoshiki S (1996) Effects of BMP-2. BMP-4. and BMP-6 on osteoblastic differentiation of bone marrow-derived stromal cell-lines. ST2 and MC3T3-G2/PA6. *Biochem Biophys Res Commun* **220**: 366–371

Yang LJ and Jin Y (1990) Immunohistochemical observations on bone morphogenetic protein in normal and abnormal conditions. *Clin Orthop* **257**: 249–256

Yoshikawa H. Rettig WJ. Lane JM. Takaoka K. Alderman E. Rup B. Rosen V. Healey JH. Huvos AG and GarinChesa P (1994*a*) Immunohistochemical detection of bone morphogenetic proteins in bone and soft-tissue sarcomas. *Cancer* **74**: 842–847

Yoshikawa H. Rettig WJ. Takaoka K. Alderman E. Rup B. Rosen V. Wozney JM. Lane JM. Huvos AG and GarinChesa P (1994*b*) Expression of bone morphogenetic proteins in human osteosarcoma: immunohistochemical detection with monoclonal antibody. *Cancer* **73**: 85–91

© Cancer Research Campaign 1998